# Ligature of the Subclavian

**Published:** 1864-02

**Authors:** 


*Ligature of the Subclavian.*—Dr. Armsby, of Albany, in November, ligated the subclavian artery. The ligature came away on the twenty-ninth day—on the FORTIETH day the patient was able to attend to business.

